# Supplementary material for: A joint role for forced and internally-driven variability in the decadal modulation of global warming
Source: Nat Commun. 2020 Jul 31;11:3827. doi: 10.1038/s41467-020-17683-7 (PMC7395113; doi:10.1038/s41467-020-17683-7)
Supplement: Supplementary file 1 — Supplementary Information [file 41467_2020_17683_MOESM1_ESM.pdf]

Supplementary Information

A joint role for forced and internally-driven variability in the decadal modulation of global warming

by Liguori[1] et al.

[1]ARC Centre of Excellence for Climate Extremes, and School of Earth, Atmosphere and Environment, Monash University, Melbourne, Australia

Modeling Center	Model Version	Model Resolution (atm/ocn)	Reference
CCCma	CanESM2	~2.8°x2.8°/ ~1.4°x0.9°	Kirchmeier-Young et al. (2017)
CSIRO	MK3.6	~1.9°x1.9°/ ~1.9°x1.0°	Jeffrey et al. (2013)
GFDL	ESM2M	~2.0°x2.5°/ ~1.0°x0.9°	Rodgers et al. (2015)
MPI	MPI-ESM-LR	~1.9°x1.9°/nominal 1.5°	Maher et al. (2019)
NCAR	CESM1	~1.3°x0.9°/nominal 1.0°	Kay et al. (2015)

Supplementary Table 1. Details on the US CLIVAR's Multi-model large ensemble simulations used in the study. Further details about the radiative forcing are listed in the table 12.1, Chapter 12 of Fifth Assessment Report of the Intergovernmental Panel on Climate Change[1]

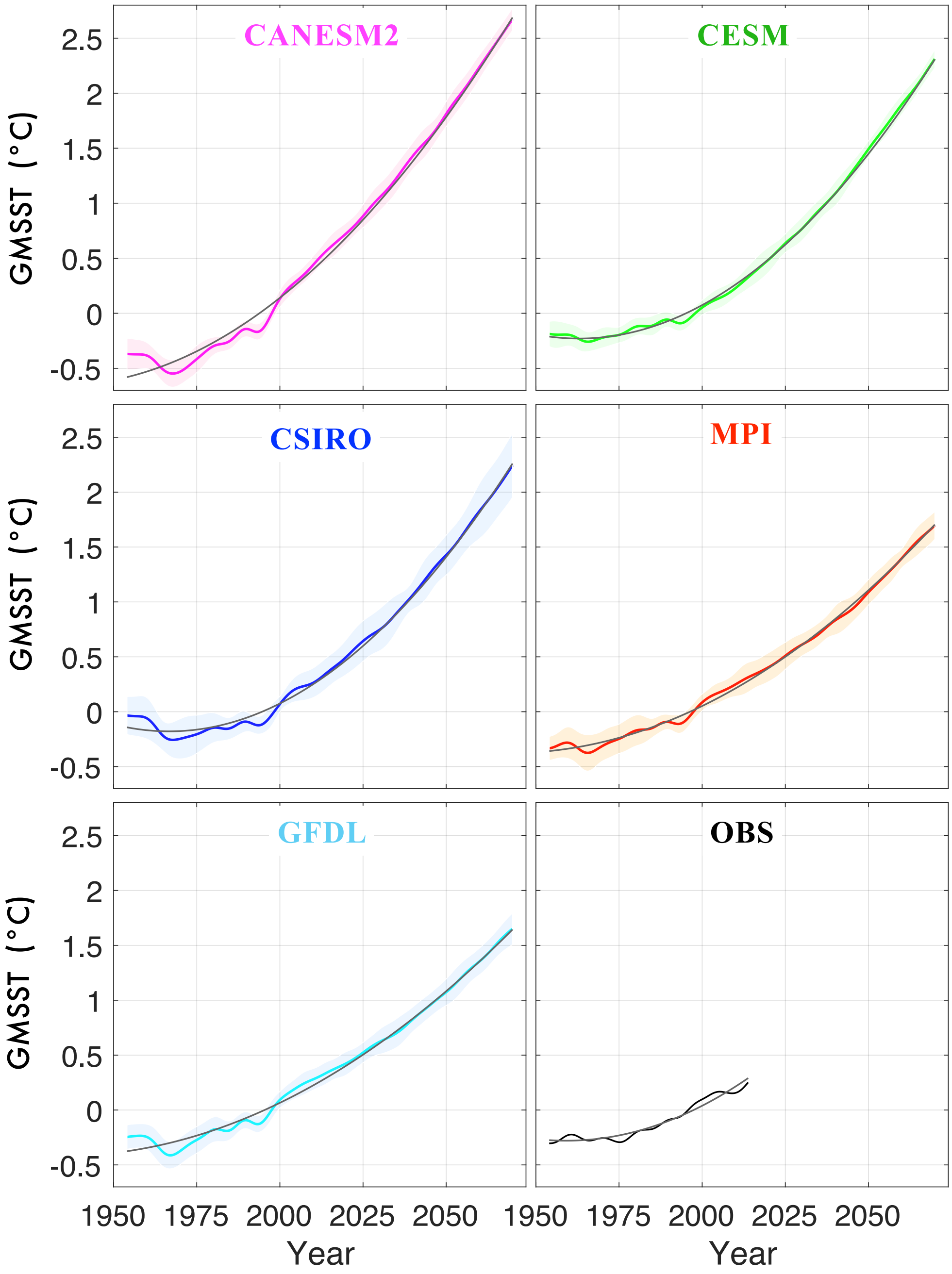

Supplementary Figure 1. Trend removal for global mean sea surface temperature. Global mean sea surface temperature anomaly (GMSST) time series with least squares quadratic fit (dark grey line) for CANESM2 (magenta), CESM1 (green), CSIRO (blue), MPI (red), GFDL (cyan) and observations (OBS; black). Colour shading indicates the two-standard deviation model spread envelope, anomalies are relative to the 1980-2010 mean, and units are in [°C].

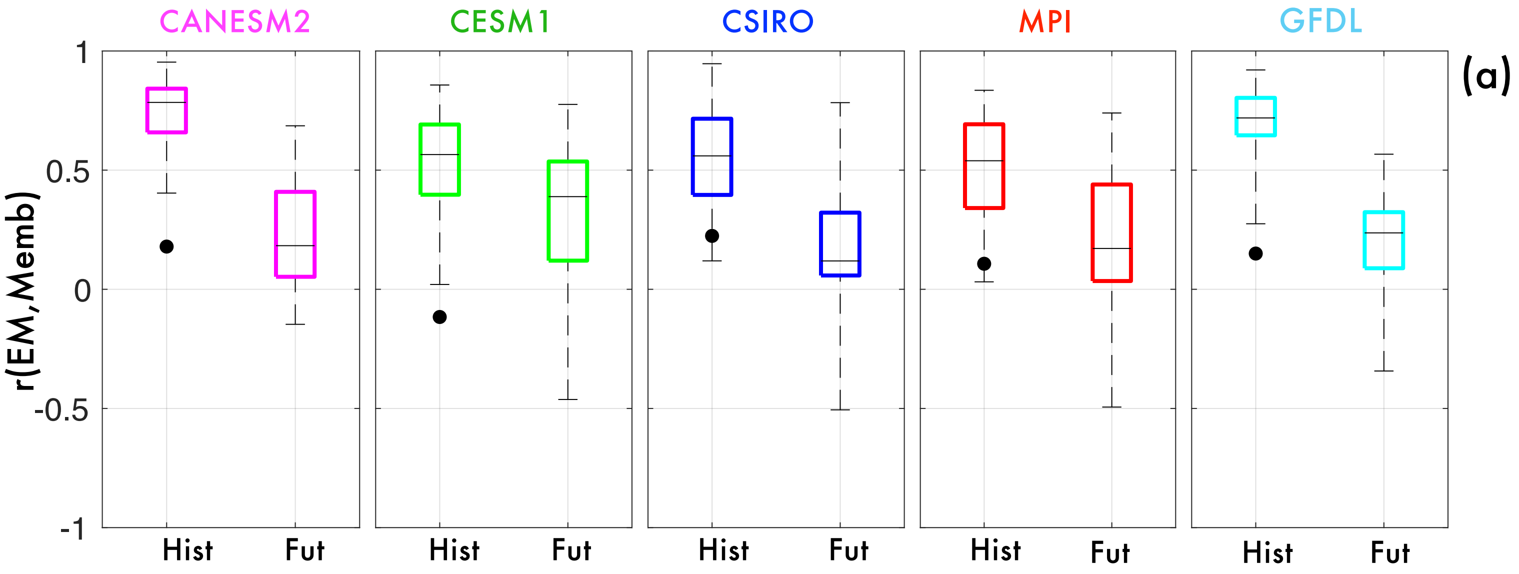

Supplementary Figure 2. Forced decadal variability and consistency with observations. Correlation coefficients (r) between ensemble mean (EM) and individual members (Memb) of the 8-year low-passed global mean sea surface temperature residual time series presented in Fig. 1b for historical (Hist) and future (Fut) periods. On each box-plot, the central mark indicates the median, the bottom and top edges of the box indicate the 25th and 75th percentiles, respectively, and the whiskers indicate minimum and maximum values. The black dot indicates the correlation between ensemble mean and observation.

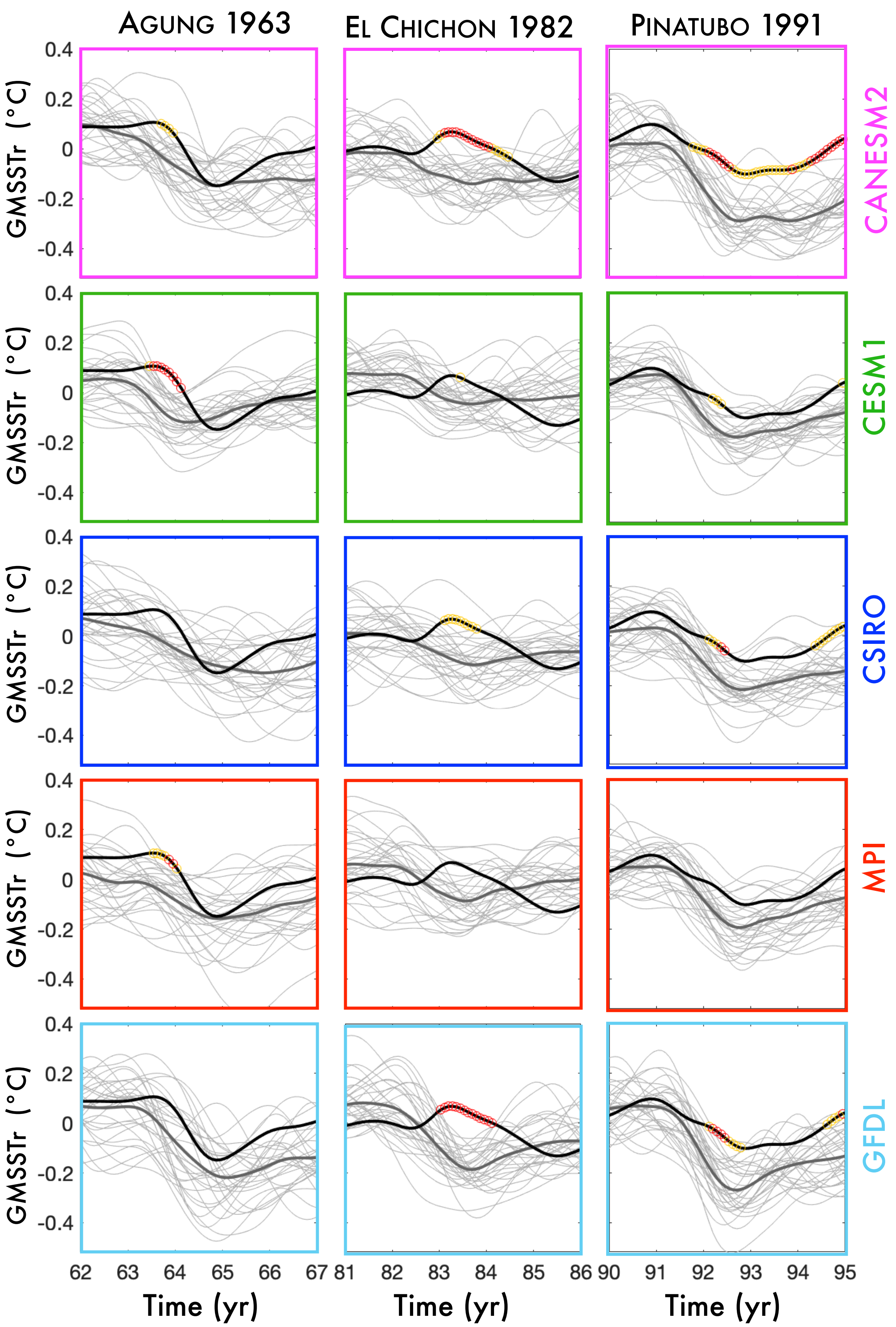

Supplementary Figure 3. Temperature response after major volcanic events. Global mean sea surface temperature residual (GMSSTr) time series used in Fig. 1c of the main text (GMSSTr obtained from 1-yr low-passed SST) during the three major volcanic eruptions of the second half of the 20th Century, namely Mt. Agung (1963), Mt. El Chichon (1982), and Mt. Pinatubo (1991). In each subfigure light grey lines indicate ensemble members, dark grey line indicates the ensemble mean, and the black line indicates the observations. Months for which the observed anomalies are higher than all (all-but-one) ensemble members are indicated with red circles (yellow circles).

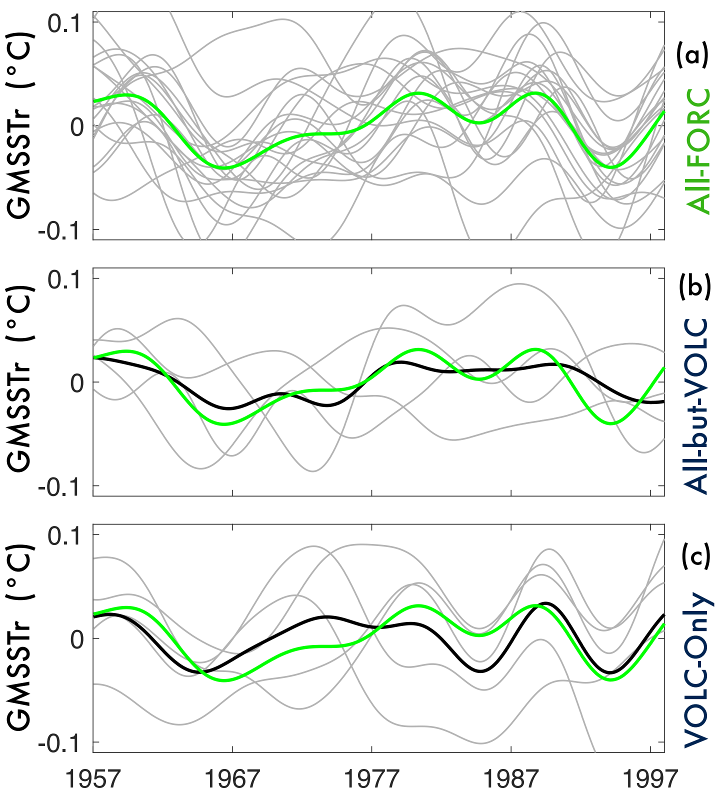

Supplementary Figure 4. Role of the volcanic forcing in CESM1. Global mean sea surface temperature residual (GMSSTr) time series in (**a**) the all-forcing ensemble (All-FORC, 20 members), **(b)** the all-but-volcanic-forcing ensemble (All-but-VOLC, 4 members), and (**c**) the volcanic-forcing-only ensemble (VOLC-only, 5 members). In each subfigures, grey lines indicate ensemble members, the black line indicates the ensemble mean, and the green line indicates the ensemble mean in the All-FORC case. Units are in [°C].

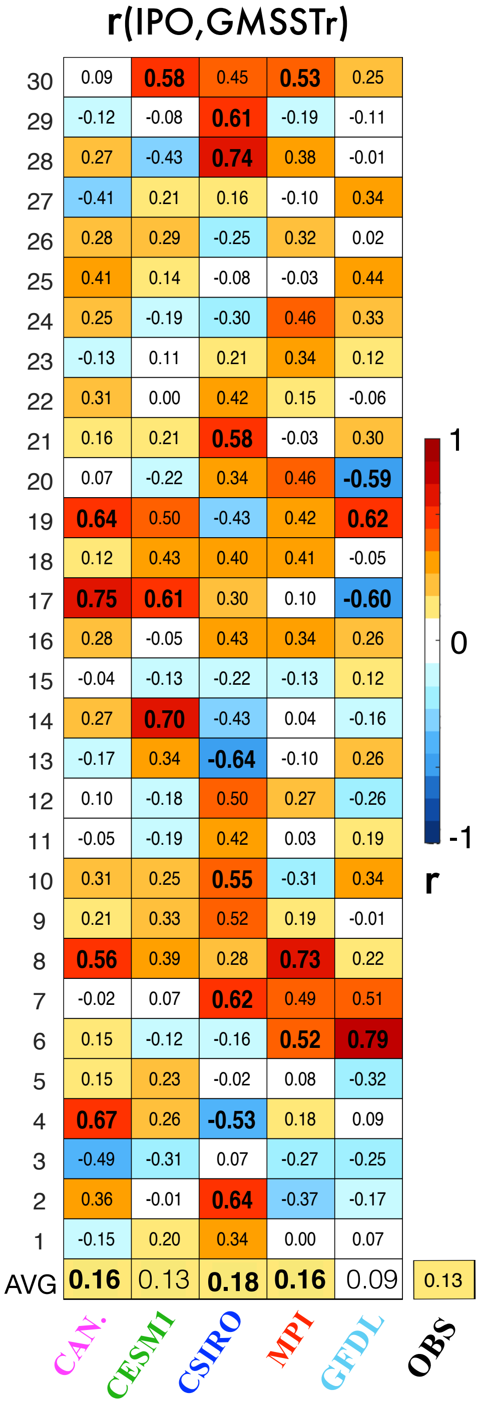

Supplementary Figure 5. Forced decadal fluctuations and Interdecadal Pacific Oscillation variability. Correlation coefficients (r) between Interdecadal Pacific Oscillation (IPO) index and global mean sea surface temperature residual (GMSSTr) time series during 1950-2010 for each ensemble member (#), the ensemble average (AVG), and the observations. IPO index is defined as 8-yr low-passed TPI index (Hanley et al., 2015). Bold numbers in indicate correlation significant at 95%. Significance levels in each ensemble have been estimated using empirical probability density functions constructed from 900 spurious correlations obtained between IPO indices during 1950-2010 and GMSSTr time series during 2010 2070.

Supplementary References

1. Collins, M., R. Knutti, J. Arblaster, J.-L. Dufresne, T. Fichefet, P. Friedlingstein, X. Gao, W.J. Gutowski, T. Johns, G. Krinner, M. Shongwe, C. Tebaldi, A.J. Weaver and M. Wehner, 2013: Long-term Climate Change: Projections, Commitments and Irreversibility. In: *Climate Change 2013: The Physical Science Basis. Contribution of Working Group I to the Fifth Assessment Report of the Intergovernmental Panel on Climate Change* [Stocker, T.F., D. Qin, G.-K. Plattner, M. Tignor, S.K. Allen, J. Boschung, A. Nauels, Y. Xia, V. Bex and P.M. Midgley (eds.)]. Cambridge University Press, Cambridge, United Kingdom and New York, NY, USA.
